# Clinical Applications of Liquid Biopsy in Prostate Cancer: From Screening to Predictive Biomarker

**DOI:** 10.3390/cancers14071728

**Published:** 2022-03-29

**Authors:** Filip Ionescu, Jingsong Zhang, Liang Wang

**Affiliations:** 1 Department of Oncologic Sciences, Morsani College of Medicine, University of South Florida, Tampa, FL 33612, USA; filip.ionescu@moffitt.org; 2 Department of Genitourinary Oncology, H. Lee Moffitt Cancer Center & Research Institute, Tampa, FL 33612, USA; 3 Department of Tumor Biology, H. Lee Moffitt Cancer Center & Research Institute, Tampa, FL 33612, USA

**Keywords:** prostate cancer, liquid biopsy, circulating tumor DNA, circulating tumor RNA, circulating tumor cells

## Abstract

**Simple Summary:**

Liquid biopsy (LB), encompassing the analysis of circulating tumor material in the blood or urine, has emerged as a powerful tool in the management of prostate cancer. In localized tumors, LB can distinguish between low- and high-grade cancers and can guide the decision to proceed with or defer tissue biopsy. In advanced disease states, LB has proven prognostic ability in addition to standard-of-care tests like the prostate-specific antigen and has been used in clinical trials to assess response. Certain LB analytes may predict resistance to androgen receptor signaling inhibitors, but how to incorporate their use into everyday clinical decision making remains unclear. Finally, for a minority of patients, LB can identify genomic alterations with significant therapeutic implications. Technological advances and creative uses of LB promise to greatly improve the management of prostate cancer patients in the near future.

**Abstract:**

Prostate cancer (PC) remains the most common malignancy and the second most common cause of cancer death in men. As a result of highly variable biological behavior and development of resistance to available agents under therapeutic pressure, optimal management is often unclear. Traditional surgical biopsies, even when augmented by genomic studies, may fail to provide adequate guidance for clinical decisions as these can only provide a snapshot of a dynamic process. Additionally, surgical biopsies are cumbersome to perform repeatedly and often involve risk. Liquid biopsies (LB) are defined as the analysis of either corpuscular (circulating tumor cells, extracellular vesicles) or molecular (circulating DNA or RNA) tumor-derived material. LB could more precisely identify clinically relevant alterations that characterize the metastatic potential of tumors, predict response to specific treatments or actively monitor for the emergence of resistance. These tests can potentially be repeated as often as deemed necessary and can detect real-time response to treatment with minimal inconvenience to the patient. In the current review, we consider common clinical scenarios to describe available LB assays in PC as a platform to explore existing evidence for their use in guiding decision making and to discuss current limitations to their adoption in the clinic.

## 1. Introduction

Prostate cancer (PC) remains a leading cause of death in the male population and the proportion of advanced cases has nearly doubled over the last decade [1]. The complexities of detecting, diagnosing and managing PC stem from its highly variable natural history with either an aggressive behavior and early visceral metastasis or, more commonly, with an indolent course marked by biochemical evidence of disease progression and subclinical osseous metastasis.

At the crux of management decisions in PC lies the balance between overtreatment and undertreatment. Current biomarkers, such as the prostate-specific antigen (PSA), or certain pathologic features (Gleason grade) render an imperfect picture of the biological behavior of PC when localized. Furthermore, the PSA is prostate-specific, but not PC-specific, and its use for screening asymptomatic individuals has been fraught with controversy. An additional layer of complexity comes from the recognition of clonal evolution as a driver of PC progression [2]. Under the selection pressure of modern therapeutics, certain malignant subclones gain a survival advantage and proliferate preferentially leading to treatment resistance and a more aggressive course. As traditional surgical pathology specimens obtained from prostate biopsies represent merely a snapshot of a decidedly dynamic process, these are inadequate for monitoring clonal evolution and may not provide necessary information to guide decision making in the advanced disease setting. The need for periodic histologic and molecular monitoring becomes apparent, but serially obtaining samples for this purpose is cumbersome because of the invasive nature of traditional biopsies and the lack of high-quality tissue for analysis when metastatic cancer is localized to the bone only. Furthermore, some patients lack any accessible metastatic lesions, have contraindications to invasive procedures or refuse repeat biopsy [3].

**Figure 1:**
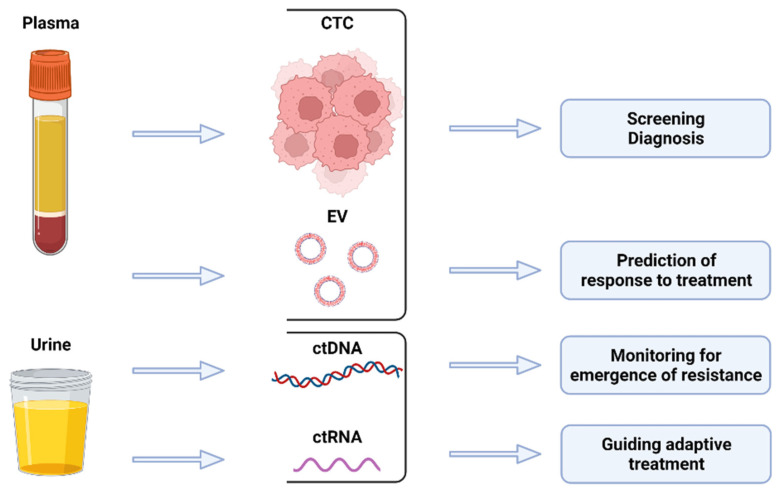
Liquid biopsy analytes and their potential applications.

As an alternative to traditional surgical sampling, liquid biopsy (LB) has emerged as a promising minimally invasive tool for PC management (Figure 1). Broadly, LB tests encompass the analysis of patient blood, urine or semen for tumor-derived material in either corpuscular form, such as circulating tumor cells (CTCs) and extracellular vesicles (EVs), or molecular form, such as circulating cell-free tumor DNA (ctDNA) and RNA (ctRNA).

From a patient perspective, the advantages of LB are primarily convenience and a more favorable risk profile. From a clinician’s perspective, LB can offer insight into disease prognosis and can potentially guide treatment decisions. Furthermore, LB can capture genomic heterogeneity that occurs randomly across metastatic sites or as a result of therapeutic pressure. Therefore, it can record the stepwise process of tumor evolution and provides the clinician with the opportunity to either prevent or respond to the development of treatment resistance [2,4].

As attractive as these novel technologies are, their adoption in the clinic continues to face several challenges. Sensitivity, especially in earlier, non-metastatic cancers, is a major limitation, as is the lack of standardization of existing methods and a paucity of high throughput methods that can directly migrate into the clinical setting. The aim of the present paper is not to provide an in-depth technical comparison of existing LB technologies or to exhaustively review all available preclinical and clinical studies investigating the use of LB in PC. We refer the reader to excellent reviews on these aspects of LB [5,6,7,8]. Instead, we summarize the current evidence for the use of LB by way of four common clinical scenarios. Where appropriate, we highlight the gaps in knowledge or technical limitations which hinder the applicability of LB. Finally, we discuss how the novel type of dynamic genomic and transcriptomic information obtained from LB may be used to guide treatment decisions in the future.

## 2. Liquid Biopsy in Prostate Cancer Screening and Diagnosis

**Case** **1.** *A 60-year-old man is seen for discussion regarding PC screening after his friend was diagnosed with metastatic disease. He is asymptomatic and has no family history of PC. He is an avid cyclist. A PSA level was checked by his primary care provider and was 4.2 ng/mL. Digital rectal examination (DRE) is normal. He is aware that the PSA is not specific for PC and that it can be slightly elevated in other diseases of the prostate. He asks whether there are any other non-invasive tests that can be performed to gauge his risk of having PC before he decides on whether or not to undergo transrectal ultrasound (TRUS)-guided biopsy.*

The issue of PC screening has been the subject of much controversy, perhaps more than any other malignancy with an available screening modality. For PC, the complexity of the matter stems both from the lack of specificity of the biomarker PSA in differentiating malignant from benign disease and from the observation that most men diagnosed with PC, depending on their age and comorbidities, will succumb to other causes [9]. For screening to be valuable in this context, it must show a significant reduction in cancer-specific morbidity or mortality if PC is detected at an early stage. Currently, the highest quality evidence available only suggests at most a small benefit in PC-specific mortality and no impact on overall mortality [10,11]. By contrast, the risks of overdiagnosis and overtreatment such as incontinence and erectile dysfunction are real and can have a substantial impact on quality of life. This observation has prompted the United States Preventive Services Taskforce to recommend selective use of PSA screening among men aged 55–69 year (grade C, offered based on clinical judgement and patient preference after a discussion of risks and benefits) and to recommend against its use in men aged 70 years or more (Grade D) [12].

The use of LB for screening purposes in PC is similarly problematic from two perspectives. First, candidate analytes are insensitive for detecting localized cancers. Both CTCs and ctDNA are either undetectable or detectable at similar rates as in biopsy-negative controls [13,14,15]. Second, in the event that future assays have improved sensitivity, there is still a concern for overdiagnosis and potential overtreatment. The experience of the DETECT-A study which used LB technology in the form of the CancerSeek platform (a combined ctDNA and protein assay) to screen for malignancy in a population of over ten thousand women can be informative [16]. While the assay detected 26 out of a total of 96 cancers (27%), two thirds of these were at advanced stages (III or IV) and, furthermore, standard-of-care screening and symptomatic presentations prompted the diagnosis of 70 out of 96 cancers (73%). This observation brings into question the clinical utility of a LB approach in cancer screening.

One established role of LB in the diagnosis of early PC is the use of the ExoDx™ Prostate IntelliScore (EPI, Exosome Diagnostics, Inc., Waltham, MA, USA), a high-throughput, urine-based EV assay which tests for the presence of the mRNA transcript of three genes (*ERG*, *PC3* and *SPDEF*) that can distinguish high-grade (≥Grade group 2) from low-grade (Grade group 1) cancer and benign tissue. The assay has been prospectively validated for a cut-point of 15.6 in men over the age of 50 years with a borderline PSA elevation (2–10 ng/mL) and, in the original study, was shown to avoid 26% of unnecessary biopsies with a negative predictive value (NPV) of 89% (overall, 7% of Grade group ≥2 would be missed) [17,18]. With this strategy, over a quarter of patients with benign disease or clinically insignificant cancer would presumably be able to avoid TRUS-guided biopsies and their respective complications (rectal bleeding, hematuria, dysuria, infection and transient sexual dysfunction) [19]. The assay is endorsed by the NCCN guidelines along with the Prostate Health Index, 4Kscore, and multiparametric MRI (mpMRI) to further define risk whenever the clinician or the patient wishes to do so before deciding on biopsy [20]. The EPI score is currently being studied in combination with other biomarkers and advanced imaging modalities, but the best way to employ these testing algorithms is unclear [21]. The SelectMDx is a similar urine-based EV assay for the detection of two mRNA transcripts for the *HOXC6* and *DLX1* genes, which are established predictors for the detection of high-grade PC and are recognized as potentially useful in certain circumstances by the NCCN guidelines [20,22]. It was validated in several prospective cohorts and has yielded NPVs in the mid 90% range and avoided roughly 35% of unnecessary biopsies while missing around 10% of high-grade cancer [23]. The precision of the assay was improved when combined with other biomarkers and mpMRI [24]. A subsequent comparison to mpMRI noted the superiority of imaging, which led to avoidance of biopsy in 49% while missing 4.9% high-risk PCs [25,26]. mpMRI with or without targeted biopsy is a powerful tool which promises to compete with or complement LB for improving the detection of early PC. Its use is well-established in patients with an elevated PSA who have had a negative systematic biopsy, a situation in which the detection rate of clinically-meaningful PC is as high as 41% [27,28,29,30]. The performance of mpMRI in selecting patients for initial biopsy is more controversial with available evidence suggesting mpMRI-targeted biopsy to be superior to systematic biopsy, but a combined approach yields the highest sensitivity [31,32,33,34]. There is limited data on how to best combine or sequence LB and mpMRI to achieve optimal sensitivity and specificity [21].

EVs are conceptually attractive targets for the development of clinically useful biomarkers. These are small vesicles released by all cells into the surrounding biofluid and, by virtue of their origin, contain genetic material (DNA, RNA), but also the end product of genetic information: protein and metabolites [35]. EVs are more abundant than CTCs and ensure the stability of their molecular contents by enveloping them in a lipid bilayer [8]. Functionally, assays analyzing EVs also confer advantages as these are continuously released by living cells (as opposed to cfDNA which is released by apoptotic cells). Preclinical data have suggested a role for EVs in the transfer of molecular material important for tumor growth and drug resistance in PC [36,37,38]. Like CTC mRNA-based assays of AR-V7, there is evidence that the identification of AR-V7 in EVs is associated with inferior progression-free survival (PFS) and resistance to enzalutamide and abiraterone [39]. While the evaluation of EV mRNA has been shown to increase sensitivity in other tumor types [40,41], small studies have cast doubt on the added value of EV mRNA-based detection of AR-V7 in PC, which was shown to be significantly less sensitive than detection in CTCs [42,43]. Preclinical studies have also suggested that EVs may play a role in the intercellular transmission of protein transcription factors or mRNA associated with taxane resistance. However, this clinically interesting question has yet to be explored in human trials. Another potential application of EVs is the detection of neuroendocrine differentiation in advanced castration-resistant PC (CRPC) [44], but clinical studies are needed. Furthermore, as metabolic reprogramming in the pathogenesis of PC becomes increasingly recognized [45], EVs may provide a snapshot of the malignant metabolic milieu as it changes with dietary or pharmacologic intervention.

Despite these promising early observations, the use of EV analysis in the management of PC outside of the two validated urine-based assays discussed above remains investigational owing to significant technical challenges. With currently available technology, the isolation and purification of EVs is suboptimal and not suitable for large scale testing [8,35,46].

One evolving area is the quantification of cell-free DNA in seminal fluid, which exhibits a higher concentration of tumor-derived DNA compared to plasma and has demonstrated significantly higher levels in PC patients compared to normal controls and patients with benign prostatic hyperplasia in small cohorts [47,48]. Further investigation of its use as a screening test and for guiding decision making in the clinic in prospective, interventional studies is warranted.

**Case** **1** **(continued).** *The ExoDx Prostate score returns as 17 and a TRUS-guided biopsy is performed which demonstrates Gleason 3 + 4 (Grade group 2) prostate adenocarcinoma in 5 out of 12 cores, consistent with favorable intermediate-risk disease. After discussion of available management alternatives, the patient opts for active surveillance.*

For patients with localized PC and very low- and low-risk disease based on the grade group, PSA level and clinical T stage, NCCN guidelines recommend active surveillance (AS) over definitive local treatment [49,50]. The decision is more nuanced for intermediate-risk disease, with AS still an option for patients with favorable characteristics of their tumor as defined by the NCCN and involves serial DREs, prostate biopsies and PSA monitoring [50]. Triggers for intervention include a short PSA doubling time, new abnormalities on DRE or upgrading on a subsequent biopsy; abnormalities on mpMRI may also form the basis for considering definitive treatment. This clinical situation is perhaps the one where LB might have the greatest impact in reducing healthcare costs and intervention-related adverse events. The most studied analyte in this setting is the prostate cancer antigen 3 mRNA transcript measured in the urine after DRE, but several adequately powered prospective trials have yielded conflicting results and this assay is not commonly utilized in practice [51,52,53,54]. One recent report identified a 3-marker urine-based panel assay consisting of *miR-24*, *miR-30c* and *CRIP3* methylation which exhibited a high NPV of 91% for reclassifying low-grade into high-grade cancers in a retrospective cohort of 103 PC patients on AS [55]. Further validation in prospective studies would be required before this panel is incorporated into clinical decision making. Blood-based assays in the AS setting are bound to have poor sensitivity given the very low levels of circulating tumor material with localized PC. However, a recent study was able to identify ctDNA in two of eight patients with localized PC using whole-genome sequencing followed by deep-targeted sequencing of selected variants in plasma cell-free DNA. In the same report, the authors demonstrated shorter metastasis-free survival in subjects with detectable pre-treatment ctDNA in a validation cohort of 189 patients [15]. The technical expertise required and the cost of the combined assay used in this analysis, however, make it unlikely that it would become readily available on a large scale in the near future.

Another scenario where LB would add tremendous value is the biochemical recurrence setting defined as an increase in the PSA following either radical prostatectomy or radiation therapy with no evidence of metastatic disease on imaging studies. Management in this clinical situation remains controversial and is rapidly changing with the advent of next generation positron emission tomography scan using highly sensitive radiotracers which may upstage patients. However, for those who continue to have PSA-only recurrence, therapeutic strategies include observation, salvage local treatment (pelvic radiation, radical prostatectomy, cryotherapy etc.), androgen-deprivation therapy (ADT) alone or ADT in combination with local therapies. To date, no clinical studies have investigated the value of LB in this scenario which, like localized PC, is unlikely to yield detectable LB analytes in circulating fluids. One can conceive, however, of improvements in the sensitivity and specificity of assays for detection of very low levels of circulating tumor material in the future and the advent of CTC-only or ctDNA-only recurrence, thus raising new questions as to the risks and benefits of earlier local salvage therapies or ADT.

Section summary: Currently, LB does not have sufficient sensitivity to perform as a PC screening test or to be used in the active surveillance of patients with established PC. LB does have a role in early PC where assays zsuch as the ExoDx™ Prostate IntelliScore can characterize the biological behavior of localized tumors and can aid in the decision to either perform or defer a tissue biopsy.

## 3. Liquid Biopsy as a Prognostic Biomarker

**Case** **2.** *A 71-year-old man with stage IV castration-sensitive PC with known asymptomatic metastatic foci in the lumbar spine and his right 4th rib is found to have additional axial skeletal lesions and one liver lesion which is confirmed prostate adenocarcinoma on biopsy. He has received external beam radiation therapy to the spinal lesions with improvement in chronic low back pain four years ago and he was treated with six cycles of docetaxel for high-burden symptomatic metastasis at that time. He is status post bilateral orchiectomy and has been maintained on enzalutamide with undetectable testosterone levels. His PSA had been stable in the 1–2 ng/mL range, but it has begun to rise over the last 6 months and has now reached 8.2 ng/mL. Liver function testing is within normal limits, and he has no symptoms related to his osseous metastases. His ECOG performance status is 0. He wishes to pursue aggressive treatment of his PC and he is offered enrollment in a placebo-controlled clinical trial of cabazitaxel in combination with an experimental Bcl-2 inhibitor, which he agrees to. Per study protocol, CTC enumeration and ctDNA level assays are obtained at baseline and results are 0 CTC/7.5mL and ctDNA is undetectable. A subsequent PSA level is checked before his third treatment cycle and is higher at 15.1 ng/mL. Concurrently, CTC and ctDNA are repeated and are unchanged. The patient asks if the elevation in the PSA level is an indication that the treatment is not working.*

PSA is a well-established biomarker in assessing response to therapy in PC. It is inexpensive, readily available and has been utilized by all clinical trials to date. It is, however, not without limitations, especially in the metastatic CRPC (mCRPC) setting where early elevations (flare phenomena) as PSA is released from apoptotic cells make it difficult to use PSA kinetics to monitor the tumor burden with sufficient precision. CTCs and ctDNA are candidate analytes to improve on the specificity of PSA flares. Stability or conversion from detectable to undetectable of these analytes is reassuring that the malignancy is responding to treatment even in the presence of a PSA elevation.

Compared to localized PC, the detection rate of CTCs in metastatic disease increases greater than fourfold [56]. The major limitation of CTC enumeration at baseline in mCRPC, however, remains the low sensitivity of detecting ≥5 CTCs/7.5 mL of blood (48–57%) [5]. CTC enumeration with the FDA-approved CellSearch platform (Menarini Silicon Biosystems, San Diego, CA, USA) has been validated in multiple prospective trials as a prognostic marker and as an early measure of response [57]. The finding of greater than 5 CTCs per 7.5 mL of blood was associated with significantly worse median overall survival (OS) and outperformed PSA as a prognostic biomarker in most studies [57,58,59,60,61]. As indicators of response, derivative measures of CTC such as the CTC0 (i.e., CTC nonzero at baseline and 0 at 13 weeks), CTC conversion (i.e., ≥5 CTCs at baseline, ≤4 at 13 weeks) or the combined CTC and lactate dehydrogenase (LDH) composite biomarker have also shown superiority compared to traditional PSA assays in assessing biochemical response and are useful in differentiating early PSA flares from treatment failure [6]. CTC enumeration may also prove useful in improving detection of disease progression as an adjunct to radiographic assessments, but studies in PC are lacking. In one analysis focused on breast cancer, CTC counts were found to be an earlier indicator of disease progression compared to standard imaging modalities, had significantly lower interreader variability (0.7% vs 15.2%) and a stronger correlation with OS [62].

Like CTCs, the level of ctDNA increases with increasing disease burden and is detected in 43%–82% of mCRPC patients with significant variation accounted for by the type of sequencing [5]. Several prospective trials and one meta-analysis have demonstrated the prognostic ability of baseline ctDNA concentration and the value of therapy-induced changes in the concentration to assess response to therapy [63]. In metastatic castration-sensitive PC (mCSPC), ADT resulted in rapid decrease in ctDNA levels in the first weeks of therapy [64]. In phase III trials performed in the mCRPC setting, baseline levels of ctDNA correlated with PFS and OS when patients received taxane-based regimens [65] or the PARP inhibitor olaparib [66]. Furthermore, a 50% or greater reduction in circulating cell-free DNA, reduction in the allele frequency of somatic mutations or complete clearance of ctDNA with olaparib treatment was associated with improved PFS and OS. Notably, the prognostic ability of a ctDNA fraction >30% remained strong even after adjustment for other clinical factors such as age, metastatic burden, LDH, PSA and hemoglobin, suggesting that ctDNA may be valuable as an adjunct to already established prognostic assays [67].

As a measure of response, LB technologies have proven more valuable in the clinical trial setting where a search for rapidly changing biomarkers can serve as surrogates for clinically relevant endpoints in a classically indolent disease.

The prognostic ability of LB in PC is well established, yet outside of infrequent situations discussed in later sections, the information derived from LB largely does not inform management decisions compared to standard of care studies. LB assays have yet to be incorporated in the RECIST criteria and, as mentioned above, the additive value of CTC enumeration to radiographic studies has not been investigated in the setting of PC. Furthermore, no prospective trials have shown that disease monitoring or early therapy switches based on results of LB assays improve patient outcomes. Such a change in therapy is not necessarily reasonable as showcased by the SWOG S0500 trial, which demonstrated the strong prognostic significance of CTCs, but found that early switching to an alternative cytotoxic chemotherapy in patients with persistently elevated CTCs (≥5 CTC/7.5 mL) after 21 days of first-line chemotherapy did not prolong OS compared to a later switch based on radiographic progression in metastatic breast cancer [68].

Section summary: Changes in CTCs and ctDNA have well-established prognostic implications independent of PSA changes and are used as markers of response in clinical trials. The additive value of these analytes to standard of care radiographic studies is unknown in PC and LB has not been incorporated in RECIST criteria.

## 4. Liquid Biopsy as a Predictive Biomarker

**Case** **3.** *A 55-year-old man with known castration-resistant PC metastatic to the lungs bilaterally, the liver and the retroperitoneal lymph nodes, complains of new pain in his chest and dyspnea on exertion over the last few months. On examination, a tender mass is palpated over his sternum. His PSA and radiographic lesions had been under control with ADT and abiraterone acetate, but current laboratory evaluation reveals a newly elevated PSA at 58 ng/mL and abnormal transaminases (AST 112 IU/L and ALT 152 IU/L). A biopsy of the sternal mass demonstrates prostate adenocarcinoma. His ECOG performance status is 2. He receives external beam radiotherapy to the sternal mass with improvement in his symptoms and completes six cycles of docetaxel with radiographic response noted in the liver metastases. He is interested in additional systemic treatment.*

Optimal sequencing of the growing number of available therapies in PC remains a contentious subject in the management of castration-resistant metastatic disease. With multiple effective drugs approved in the first line, ranging from several novel androgen receptor signaling inhibitors (ARSi) and taxane-based chemotherapy to immunotherapy and radiopharmaceuticals, the choice is often guided by symptomatology, location and burden of metastatic disease, as well as patient fitness. Beyond the first line setting, prior therapies and symptomatology weigh the most in subsequent treatment choice.

Numerous studies have suggested that identification of androgen receptor (AR) aberrations such as amplification, point mutations, rearrangements and splicing variants leading to reactivation of signaling are present in 50–70% of advanced PC patients and are associated with worse outcomes in mCRPC treated with ARSi [69,70,71].

The most common genomic aberrations identified in PC are AR copy number variations (CNV), specifically AR amplification, and AR point mutations [67,72,73,74,75,76,77,78,79]. A meta-analysis of 16 studies representing more than 1000 patients found that AR gain is associated with a shorter PFS and OS in patients treated with ARSi, but it had no effect in patients treated with docetaxel in the first line or cabazitaxel in the second or third lines [80]. Apart from AR gains, amplification in the enhancer region upstream of the AR gene has been found to be more prevalent (present in up to 80% of mCRPC) and outperformed AR-V7 expression in its prognostic ability [70,81,82,83]. Point mutations in the ligand-binding domain of AR generally result in either resistance to ARSi’s or receptor promiscuity (activation by corticosteroids or female sex hormones) [71]. These occur in approximately 20% of all mCRPC and the frequency of specific resistance-conferring alterations appears related to which ARSi a patient was exposed to [84]. For example, the promiscuous L702H mutation is frequently seen following abiraterone acetate treatment [2,85]. Other mutations such as T878A and H875Y result in AR activation by second generations ARSi’s and are associated with shorter PFS [85,86]. In vitro studies have suggested ways to mitigate resistance to certain point mutations. One study found that the L702H AR mutant is activated by prednisone and cortisol, but not dexamethasone [84]. Several analyses have demonstrated the ability of darolutamide, a second-generation ARSi, to inhibit the transcriptional activity of enzalutamide-resistant mutant ARs (F877L, H875Y/T878A, F877L/T878A) [87,88]. The real-world implications of these findings remain to be explored. Importantly, AR amplifications and point mutations are detected by commercially available ctDNA assays and have been shown to be concordant with aberrations seen in matched metastatic tissue [89].

Perhaps the best characterized splice variant is AR-V7, a constitutively activated isoform of the AR, the presence of which may be helpful in the process of choosing between chemotherapy and novel ARSi’s in the frontline setting in mCRPC [90,91,92,93]. AR-V7 testing can be performed on genetic material obtained from CTCs or using circulating genetic material such as ctRNA. Nuclear localization of the AR-V7 protein product can be demonstrated using a CTC-based immunofluorescent assay and is commercially available as the Oncotype DX AR-V7 Nucleus Detect^®^ test (Genomic Health, Redwood City, California) [92,93]. Multiple prospective studies and two recent meta-analyses have reported on the prognostic ability of AR-V7 with most analyses demonstrating an association with decreased PFS and OS in mCRPC patients treated with ARSIs, but not with taxanes [94,95]. These observations have prompted some authors to suggest that detection of the AR-V7 should prompt use of taxanes over ARSIs as first line treatment for mCRPC [96].

Cost-wise, the ctDNA assays and the Oncotype DX AR-V7 Nucleus Detect^®^ test are similar, but the former provides information regarding genomic alterations beyond those involving the AR, such as actionable mutations in the DNA repair pathways which are described in a later section. For this reason, ctDNA assays are more attractive and theoretically more likely to inform clinical practice.

The utility of AR aberrations to guide treatment in the first-line setting is limited by their low prevalence in populations that have not been exposed to abiraterone, enzalutamide or taxanes [76,85,97,98]. With increasing use of these agents in mCSPC patients, their role might become better defined in the future, but to date, there are no prospective trials investigating the prognostic or predictive ability of AR aberrations in PC. Furthermore, as high-quality evidence comparing first-line chemotherapy to ARSis are lacking, decisions in the clinic are based primarily on patient characteristics. Generally, for symptomatic patients and those with high-burden or visceral disease who are otherwise fit, the use of chemotherapy in the first line is reasonable as this approach has a demonstrated OS benefit and improved palliation regardless of the presence of AR aberrations [96,99,100]. Conversely, for patients with asymptomatic and indolent disease, the decision between first-line options is more nuanced as quality data comparing existing agents is scarce.

LB may be most likely to impact selection of second- and later-line agents in the mCRPC disease state as the rate of detectable genomic and transcriptomic aberrations increases as a result of therapeutic pressure [2,85,97,98]. After progression on first-line docetaxel, options include cabazitaxel, abiraterone and enzalutamide, which all have shown response rates in the 26–37% range in individual analyses [101,102,103,104]. Demonstration of AR aberrations as a marker of ARSi resistance could potentially guide a physician towards a taxane-based regimen which has been proven to maintain activity in this setting and the use of AR-V7 expression is suggested by the current NCCN guidelines to aid with this decision [50]. Notably, the recent CARD trial demonstrated superiority of cabazitaxel in the third-line setting over the alternative ARSi in men who had progressed on docetaxel and an ARSi [105]. As the study did not report genomic data, it remains unclear if patients who lack AR aberrations after failing an ARSi may still respond to the alternative ARSi.

From a practical perspective, however, it may be reasonable to trial the alternative ARSi in this setting, especially if LB does not identify resistance-conferring AR aberrations, given the ease of administration and favorable side effect profile of these agents. Indeed, alternating between ARSi agents remains a prevalent practice pattern [92]. Despite the concern for cross-resistance [106,107,108,109], activity of an alternative ARSi is still possible in the second line, especially with the abiraterone → enzalutamide sequence. After abiraterone failure, multiple retrospective, real-world studies have found biochemical response rates to enzalutamide in the range of 10–40% and to docetaxel in the range of 25–40% [101]. One prospective analysis concluded that a trial of enzalutamide after abiraterone can still provide benefit in up to 27% of patients [110]. By contrast, the enzalutamide → abiraterone sequence results in response rates of only 2–8% [101].

Real-world data suggests that LB is becoming an adjunct to clinical decision making. A cross-sectional, questionnaire-based study surveyed 150 clinicians in the United States and Canada on their use of AR-V7 testing and found that AR-V7 positivity changed management in greater than half the patients (62%), who were preferentially treated with taxane-based regimens (43%) or were referred for a clinical trial (43%) [111]. The physician-reported biochemical response rate was also significantly higher in those patients who had a change in treatment based on AR-V7 positivity (54% vs. 31%). A cost analysis from the same group suggested that this testing strategy can prove cost-saving [112].

Serial AR-V7 testing may also be of value as taxane-based therapy can cause a reversion of AR-V7 positive to negative status in approximately one third of patients [113]. In this situation, rechallenging with ARSi may be reasonable, but the clinical benefit of this approach in patients who have converted to AR-V7 positive status after progression on first-line ARSi and revert to an AR-V7 negative status after taxane-based therapy is unknown. Nonetheless, implementation of serial testing for genomic aberrations in adaptive therapy strategies is an attractive area of study.

Despite these encouraging early reports, the added value of AR aberrations must still be considered primarily prognostic, as no prospective interventional trials have compared patient-specific outcomes in cohorts treated as guided by LB and those treated per physician preference beyond the first-line setting. However, the predictive role of AR aberration detection is bound to become more important with the advent of novel therapeutics such as niclosamide, TAS3681, ARV-110 and JNJ-63576253, which degrade or antagonize mutated or variant AR and are currently investigated in phase I/II trials (NCT02807805, NCT03123978, NCT02566772, NCT03888612, NCT02987829). Furthermore, two ongoing precision medicine trials with an umbrella or platform design are actively utilizing ctDNA to select patients which have progressed on ARSi for molecularly matched agents such as inhibitors of CDK4/6 (palbociclib), WEE1 kinase (adavosertib), cMET (savolitinib), the AR inhibitor darolutamide, carboplatin or immune checkpoint inhibitors (NCT03385655, NCT03903835).

**Case** **4.** *A 52-year-old male with mCRPC with adenocarcinoma histology is seen for a second opinion. He has known bone metastases involving the thoracic and lumbar spine, as well as involvement of several retroperitoneal lymph nodes causing right ureteral obstruction which have necessitated percutaneous nephrostomy. In addition to ADT, he has been treated with abiraterone, enzalutamide, Radium 223, docetaxel and cabazitaxel. Recent staging imaging revealed additional bone metastases in the proximal femurs and new liver lesions. Laboratory studies are remarkable for alkaline phosphatase and transaminase elevations to 3 times the upper limit of normal. Clinically, he has mild back pain which is controlled with over-the-counter medication. He is still able to work as an accountant. He is interested in additional treatment.*

One established use of LB is the identification of patients with actionable genetic alterations in patients who have progressed despite established first-, second- and third-line therapies. Frequently, these arise under therapeutic pressure late in the disease course making evaluation of the original biopsy specimen inadequate for their detection. Furthermore, unlike the patient described in Case 4, many times metastatic disease is localized exclusively in the bone or involves difficult to biopsy lymph nodes in the retroperitoneum.

The emergence of visceral metastasis, however, presents a different diagnostic dilemma with the possibility of transformation of prostate adenocarcinoma to a small cell phenotype. This process, termed neuroendocrine (NE) transdifferentiation, occurs in up to 17% of treatment-resistant mCRPC [114], currently requires a surgical biopsy for diagnosis, and has important prognostic and therapeutic implications. Although not commercially available, LB assays have recently shown promise in this space. Strategies for the non-invasive detection of NE transformation include identification of a neuroendocrine CTC phenotype (using immunofluorescent staining for DAPI, CK, CD45, and AR, as well as cytoplasmic and nuclear characteristics), single CTC analysis showing the combined loss of tumor suppressors RB1, TP53, and PTEN or expression of AR-v567es, and ctDNA analysis demonstrating concurrent genomic (TP53, RB1, CYLD, AR) and epigenomic (hypo- and hypermethylation of 20 differential sites) alterations [115,116,117,118].

In advanced cases of treatment-refractory prostate adenocarcinoma, precision oncology has identified several mutations with implications for late-line treatment options [119]. The best studied are alterations in DNA repair genes involved in the homologous recombination (HR) pathway (somatic or germline mutations BRCA 1, BRCA 2, ATM) present in up to 33% of advanced mCRPC patients and conferring sensitivity to PARP inhibitors and platinum agents [120]. Notably, the prevalence of HR aberrations increases under therapeutic pressure as demonstrated by a large retrospective study which found a prevalence of 27% in metastatic samples, but of only 10% in primary tumors [121]. This finding underscores the importance of serial genomic monitoring. Mutations in mismatch repair genes (MLH1, MSH2, MSH6, PMS2) are less common and are encountered in up to 5% of prostate adenocarcinomas but have important clinical implications. Like the HR genes, these mutations can occur in the germline or can be acquired somatically and their prevalence increases under therapeutic pressure in advanced stages [122]. Mismatch repair-deficient tumors are characterized by microsatellite instability (MSI high [MSI-H]) and development of neoantigens, which confer susceptibility to the PD-L1 inhibitor pembrolizumab. Mutations in the transcription factor regulating the expression of DNA repair genes, CDK12, have also been shown to predict response to immunotherapy [123]. Several randomized trials have shown biochemical and radiographic responses, as well as improvement in PFS with PARP inhibitors in advanced prostate cancer [124]. The use of pembrolizumab in MSI-H PC led to objective responses in small case series and retrospective analyses [125,126,127].

There are many commercially available next generation sequencing (NGS) assays which have been FDA-approved in all solid malignancies to detect mutations in DNA repair genes and CDK12. For PC in particular, FoundationOne^®^Liquid CDx is validated for the detection of BRCA1 and BRCA2 alterations to identify candidates for the PARP inhibitor rucaparib.

Serial monitoring has also been shown to detect reversal of BRCA mutations to wild type alleles which can also occur under therapeutic pressure with PARP inhibitors or platinum agents [66]. Although not as well characterized, one analysis found that disappearance of MSI from blood correlated with biochemical and radiographic response [127]. These observations suggest that serial LB may prove useful not only as a positive predictor of response to treatment in advanced disease states, but also for identifying patients in whom continuing certain agents is futile.

Section summary: Most PC patients will eventually develop aberrations in the AR as a result of therapeutic pressure. These are detectable by commercially available LB assays, have prognostic implications and predict resistance to novel ARSi therapy. How to best incorporate information on AR aberrations into clinical practice remains controversial as no interventional trials to date have proven that LB-guided changes in treatment from ARSi to taxane-based regimens improve patient outcomes. LB can also detect mutations in the DNA repair genes (MMR and HR pathways) which arise in a minority of patients and have distinct therapeutic implications.

## 5. Current Challenges for the Incorporation of Liquid Biopsy in the Management of Prostate Cancer

**Figure 2:**
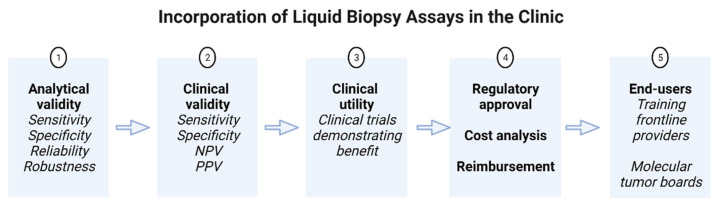
Steps in the incorporation of liquid biopsy assays in clinical practice. NPV, negative predictive value; PPV, positive predictive value.

Challenges in incorporating LB in patient management can arise at several levels: assay performance, regulatory organizations or the clinician end user (Figure 2) [7].

The Evaluation of Genomic Applications in Practice and Prevention (EGAPP) initiative identifies three conditions which must be met for validation of any assay before it can be included in clinical practice: analytical validity, clinical validity and clinical utility [128]. Analytical validity is defined as an assay’s ability to reliably and accurately measure a specific analyte with appropriate analytical sensitivity, specificity and robustness. Clinical validity, by contrast, characterizes the accuracy of the assay in a clinical setting and is described in terms of clinical sensitivity, specificity and negative and positive predictive values. Currently, the sensitivity of LB remains low and, in the search for actionable mutations, there is consensus that a negative LB test should be reflexed to tissue testing [7]. The specificity of LB is also threatened by the co-occurrence of similar mutations in ctDNA and hematopoietic cells as a consequence of clonal hematopoiesis of indeterminate potential, a phenomenon more prevalent in the aging population. This overlap in the genomic profiles of PC and clonal hematopoiesis, when mutations in ATM, BRCA2 and CHEK2 are detected, may lead to incorrect identification of older patients as candidates for PARP inhibitors when these aberrations are not in fact present in the PC genome [129]. One particular challenge to LB sensitivity is the detection of CNV, the most frequent genomic aberration in PC, in samples with a low content of ctDNA where the larger fraction of free DNA derived from non-cancer cells almost completely masks the signals from cancer cells [130,131]. Recent advances in assay sensitivity and bioinformatic processing, however, have improved upon the performance of LB detection of CNV [130,132].

Many LB assays that have shown promise in PC remain primarily research tools accessible to a small community with special expertise in carrying them out. There is great heterogeneity amongst laboratory approaches, reagents and equipment used and no comparison studies have been performed, making inferences regarding the analytical validity of any given method on a large scale difficult. The concern about inter-assay concordance extends to high throughput, commercially available tests as well, which have shown significant variability as a result of technical factors (bioinformatic processing, background noise, filtering cut offs) especially for low variant allele frequencies [133,134]. Furthermore, because of their often laborious and operator-dependent nature, many research methods are seldom amenable to becoming high throughput tests.

Perhaps the most evident limitation for incorporating LB in routine decision making is the lack of high-quality evidence to support its clinical utility in PC management. As outlined above, although the number of prospective trials in PC is increasing, all were observational in nature. No interventional trials have demonstrated a benefit in changing management based on results of LB assays in PC and until these are available the interpretation of these tests and how they guide decisions in the clinic must be done with caution. The final steps in scrutinizing the assays themselves, after meeting criteria for analytical validity, clinical validity and clinical utility, are the rigorous approval process by regulatory organizations, cost analyses, inclusion into treatment guidelines, and approval for reimbursement by payers.

Beyond the barriers outlined above, there are also important end user-related factors that play an important role in incorporating LB in the clinical workflow. As assays become more specialized, testing will be increasingly carried out in central laboratories that have the necessary expertise and resources. Most contemporary commercial sequencing assays are already conducted in central laboratories with access to advanced NGS machines, technicians for direct lab work, as well as multidisciplinary teams comprised of biologists, clinicians and bioinformaticians that can process the large amount of genetic data and translate it into clinically actionable information. The advantage of centralized testing, beyond that of technological resources and expertise, is economical, as these centers frequently serve institutions nationally or internationally and can decrease costs through economies of scale. By contrast, tests which are performed locally, such as the CellSearch platform for CTC enumeration, may not be as cost- or labor-effective. The major disadvantage, however, of centralized testing is that it results in a lack of bidirectional communication between the frontline provider making the decision and the committee interpreting the lab assay. In short, there is no direct clinical integration of the individual patient situation. As a response, many larger centers have created their own multidisciplinary precision oncology tumor boards, where pathologists, radiologists and clinicians directly discuss patient cases while considering the results of NGS panels obtained on tissue or ctDNA. Still, most institutions will not have access to such specialized teams, highlighting the need for educational initiatives targeting frontline providers, ranging from phlebotomists and lab technicians who are involved with initial processing of the LB specimen to clinicians who will have to read the report, interpret the result and correctly integrate the information into their clinical decision-making process.

## 6. Future Directions of Liquid Biopsy Assays in Prostate Cancer

Efforts to optimize LB are underway on several different fronts. The growing use of droplet digital polymerase chain reaction (ddPCR) technologies promise to increase assay sensitivity (up to 90% in some studies focused on lung and colorectal cancer) and, by contrast to previous generations of PCR, are able to directly quantitate molecular LB analytes making it possible to reliably track analyte levels in response to treatment and to monitor for development of resistance [135,136,137]. Similarly, non-PCR-based technologies for ctDNA quantitation such as fluorimetry (Qubit) may improve the turnaround time and cost of LB assays while preserving a similar sensitivity to that of quantitative PCR which represents the current gold standard method [138,139].

In line with the evolution of tissue biopsies where immunohistochemistry, cytogenetics, single gene assays and multi-gene NGS are used to complete traditional morphologic examination, combinations of LB analytes are emerging as an important modality to increase test accuracy and construct a more complete genomic profile. Particularly, combining genomic and transcriptomic analysis represents an attractive area of investigation. From a biological perspective, integrating transcriptomic information derived from ctRNA has several theoretical advantages [140]. First, the expression of RNA tends to change as an adaptation to the internal needs of the malignant cell, whereas DNA is largely stable across a malignant cell clone. Second, RNA-based approaches permit detection of fusion transcript, splice variants and non-coding nucleic acid molecules that may have clinical implications or be specific to a particular tumor phenotype. Furthermore, it is becoming clear that the molecular biology of PC is not entirely dictated by mutations in DNA, but rather the product of both genetic and epigenetic changes. It is important to note that the only assay that can detect clinically significant PC non-invasively is the urine-based EPI score which tests for three RNA gene signatures, whereas no ctDNA aberrations have been shown to reliably do so [17,18]. As a further proof of concept, one of the most studied candidate predictive biomarkers is the AR-V7 transcript which is a product of alternative splicing detectable only in RNA or its respective protein product. Transcriptomic analysis of certain AR-related gene clusters has also been recently shown to provide additional prognostic value independently of AR-V7 [73].

Results from studies using multiparametric LB assays highlight the complementary nature of concomitant ctDNA and ctRNA analysis. Concurrent genomic and transcriptomic aberrations (AR amplification and the presence of AR splicing variants) have been associated with worse biochemical, clinical and radiographic PFS compared to any other single AR aberration identified in either ctDNA or ctRNA; the impact of these composite analytes on overall survival has been less clear [74]. Attempts to investigate the impact of combined CTC enumeration and either ctDNA or ctRNA on prognosis have not been as promising, but the addition of circulating nucleic acid analysis to CTC enumeration, however, can theoretically improve the sensitivity of an assay given the relative rarity of CTCs and the greater technical difficulty in sequencing CTC-derived genetic material [74,75]. A pilot study simultaneously analyzed ctDNA, ctRNA, CTC DNA and germline DNA as part of a multiparametric LB assay in advanced PC patients and demonstrated genomic alterations that were common between CTC DNA and ctDNA, but also alterations that were uniquely identified in only one of the two analytes [141]. The authors made special note of the challenge of compiling and interpreting the large amount of genetic information generated by the combined assay. Overall, it remains unclear how the presence of one genetic abnormality in one LB analyte and not in another translates into clinical decision making. As genetic information obtained from LB becomes more abundant with more sensitive or combined assays, it is paralleled by a growing need for rationally designed, prospective interventional trials addressing real-world clinical questions.

**Figure 3:**
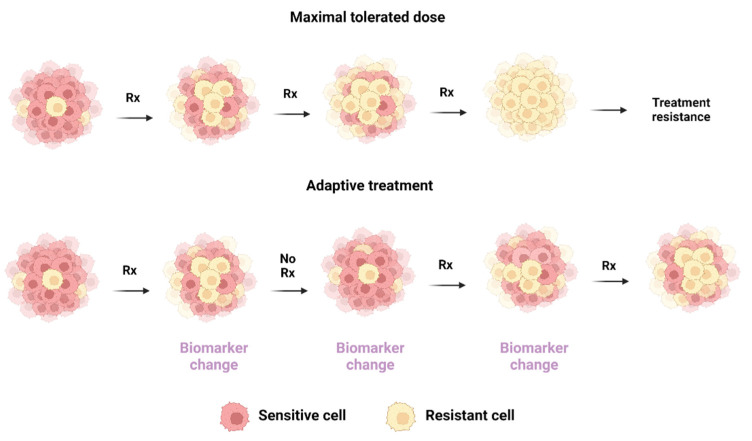
Comparison of maximally tolerated dose and adaptive treatment strategies utilizing liquid biopsy. Rx, treatment.

Apart from technological advancements, novel uses of LB assays are also being explored, particularly as candidate biomarkers in the emerging field of adaptive cancer therapy. Drawing from evolutionary biology and game theoretical principles, adaptive therapy uses mathematical modelling to challenge the prevalent therapeutic paradigm of using maximally tolerated doses (MTD) of active treatments until progression [142]. PC tumors are comprised of heterogenous cell populations with distinct susceptibilities to standard of care antiandrogen treatments and the proportion of each subpopulation has been shown to change as a result of therapeutic pressure [2,143]. In this context, an MTD strategy can lead to more rapid selection of resistant subpopulations. By contrast, an adaptive treatment strategy aims to prolong treatment sensitivity by maintaining a balance between competing malignant subpopulations while controlling tumor size. It does so by strategically withdrawing the therapeutic agent and allowing the sensitive population to proliferate, in turn inhibiting the growth of resistant clones. The process is illustrated in Figure 3.

Mathematical models of adaptive treatment strategies have been developed for mCRPC and the feasibility, safety and efficacy of this approach have been investigated in mCRPC with promising results [142,144,145,146,147,148]. In a pilot study using a 50%-change in the pretreatment value of PSA as a biomarker to guide initiation or withdrawal of abiraterone, the median time to treatment failure defined as radiographic progression was as long as 27 months with an adaptive strategy, representing a substantial increase compared to 16.5 months with continuous treatment described in the original studies [142,149]. PSA, however, represents only a surrogate marker of tumor burden and utilizing LB to detect the emergence of certain genomic or transcriptomic aberrations (AR amplification, AR-V7, AR point mutations etc.) may signal the development of resistance earlier during treatment or at lower burdens of the resistant subpopulation and, thus, improve the performance of such adaptive models. Furthermore, the real-time detection of AR amplification and AR-V7 as demonstrated by LB analytes are currently thought of in dichotomous terms with their presence indicating resistance and their absence indicating sensitivity to ARSi. In the adaptive treatment paradigm, quantitation of these analytes can be conceptualized on a spectrum of resistance and serial monitoring of on-treatment levels with LB can guide clinical decisions of strategically withdrawing anti-anadrogen treatment or switching to cytotoxic chemotherapy.

## 7. Conclusions

LB is an exciting novel technology that is slowly making its way into clinical practice. Despite the availability of several powerful, high throughput tests, the clinical scenarios where it can inform management beyond simple prognostication in PC remain few. The main limitation to the incorporation of LB in clinical decision-making is the absence of prospective interventional studies that demonstrate improved patient outcomes with LB-directed therapeutic changes. Thus far, the ever-increasing amount of genomic and transcriptomic data yielded by LB has made it challenging to separate the signal from the noise and constructing rational trials has been a moving target. As our understanding of how to incorporate knowledge of numerous concurrent molecular aberrations evolves, information will become more easily translated into clinically actionable steps. Looking forward, improvements in LB sensitivity, multiparametric testing and the creative new applications of LB for tracking clonal evolution and devising adaptive treatment strategies, promise to revolutionize PC care in the coming years.
